# The Treatment of Pre-Tuberculous Stage of Consumption

**Published:** 1914-01

**Authors:** 


The Treatment of Pre-Tuberculous Stage of Consumption. Alfred S. Gubb, M. D., L. R. C. P. Lond., M. R. C. S. Eng., D. H. P., etc., Aix-le-Baines, Savoie, France (*Medical Herald*). Except for the discovery of the bacillus of tuberculosis, the most interesting outcome of recent research has been to show that the germs of tuberculosis will only grow on suitable soil, that is to say, soil which has been prepared for infection by inherited or acquired debility. It is this stage of liability to infection that constitutes the so-called pre-tuberculous period, the investigation of which has revealed several interesting facts.

Thanks in a great measure to Professor Albert Robin of Paris, who made a special study of the physiological features of this pre-tuberculous period, we know that it is characterized by a curious but striking instability of the mineral constituents of the tissues, notably the chlorides and phosphates. This tendency to phosphaturia of course is by no means peculiar to tuberculosis for in a more or less fugitive form it is met with in many morbid states, from simple dyspepsia to albuminuria. The distinguishing character of the leakage of phosphates occurring in connection with tuberculosis is its constancy. It is this constancy that constitutes its gravity, because, in the long run, it determines pronounced impoverishment of the tissues in respect of their mineral constituents.

It would be rash to assume forthwith that the amenability of the tissues to tuberculous infection is the direct, inevitable consequence of this loss of phosphates, because the inability to hold and to retain the mineral elements may, after all, be merely an outward and visible effect of the same vital weakness that creates the proneness to infection, just as the loss of appetite determines a state of debility that predisposes to infection from lack of nourishment.

However produced, and whether due to an inherited inability of the tissues to maintain their nutrition or to the disturbing influence of chronic intoxications and other causes of organic debility, the persistent phosphatic waste engenders a state of malnutrition that places the organism in a manifest condition of inferiority.

The recognition of this predisposing process affords a clear indication for treatment, and the measures that have for their object the remedying of this source of debility and the cutting short of the pre-tuberculous stage constitute the prophylactic treatment of consumption. Just as drainage and the application of lime to an impoverished land wards off mildew and blight that attack imperfectly nourished vegetables, so hygienic measures and the administration of lime salts to persons who are threatened with consumption tend to enable the tissues to resist their natural enemies.

That this is no mere theoretic conception is shown by the comparative ease with which threatened consumption, and even the incipient stage of the actual disease, can be averted or cured by appropriate treatment. Remove the cause, said Hippocrates, and the effect will disappear, and in most instances it is possible to remove the cause of the predisposition to phthisis.

But before we discuss the treatment there is another physiological factor that calls for notice, namely, the persistently low arterial tension. So constant is this low blood pressure that it is now regarded, in the absence of any other explanation, as diagnostic of impending consumption. A young man apparently in the enjoyment of a fair standard of health, whose blood pressure is persistently below 110 millimeters should be looked upon with suspicion, although for the time being there may be no signs of pulmonary mischief accessible to the stethoscope.

The two principal features of the pre-tuberculous stage of pulmonary tuberculosis are, therefore, increased elimination of phosphates and a persistently low blood pressure.

Other disturbances of the vital processes have been noted—changes in the respiratory quotient, for instance—which likewise possess grave significance, but we need not dwell upon these, seeing that they have no direct bearing on treatment.

Inasmuch as the phosphatic waste may conceivably be due to tissue debility, it behooves us to place the organism under conditions favorable to its recuperation, and these may be summed up in the therapeutical trinity: fresh air, good food, and rest. These alone, however, may not suffice to restore the nutrition of the tissues. There is lost ground and arrears of nutrition to be made up, and it is asking too much of the jaded organism to expect it to "pay in advance," that is to say, not only to secure the adequate nutrition of the tissues which it has so far been unable to obtain, but also to restore the debit balance created by past depredations.

Medicinally the plan of campaign is already traced. We are called upon to make good the phosphate waste (and incidentally the chloride waste as well), and to stimulate the processes of nutrition by raising the blood pressure to a higher level. Higher arterial tension means freer irrigation of the tissues, freer irrigation of the tissues, in its turn, means improved nutrition. Now lime and strychnine both tend to raise the blood pressure, and if they be given in the form of phosphates, all the therapeutical indications will have been fulfilled.

Some phosphorous compounds, however, are more readily assimilated than others, and for this reason it is better to employ the hypophosphites. In order to facilitate their apprehension by the tissues the phosphorus should be administered in combination with various bases, and advantage may be taken of the opportunity to introduce medicinal tonics, such as quinine, iron, manganese, potassium, etc. Such a compound is presented in the well-known Fellows' syrup, which must have been devised in deference to the principles enunciated above, and has justified its existence by the results that follow its employment.

Its success, in all probability, is due to the fact that, by enhancing arterial tension, it enables the tissues to avail themselves of the accompanying phosphorus salts and so to reconstitute their nutrition. Under its influence, indeed, the subject gains in weight while his digestion, in common with the other vital functions improves; and the taste of physiological misery gradually disappears.

